# Short-term effects of tillage and residue on spring maize yield through regulating root-shoot ratio in Northeast China

**DOI:** 10.1038/s41598-017-13624-5

**Published:** 2017-10-17

**Authors:** Debao You, Ping Tian, Pengxiang Sui, Wenke Zhang, Bin Yang, Hua Qi

**Affiliations:** 1 College of Agronomy, Shenyang Agricultural University, Shenyang, 110866 Liaoning China; 2 Key laboratory of Tropical Forest Ecology, Xishuangbanna Tropical Botanical Garden, Chinese Academy of Sciences, Menglun, Yunnan 66303 China

## Abstract

In recent years, yield instability of spring maize becomes increasingly pronounced under the traditional cropping system. In 2014 and 2015, short-term effects of tillage (plow-till, rotary-till and no-till) and residue (removal and incorporation) on soil properties, maize growth and yield were investigated in a brown soil region. Our results indicated that short-term reduced tillage (rotary-till and no-till) and residue incorporation promoted soil properties and maize growth. Compared with plow-till, rotary-till and no-till decreased soil bulk density and compaction below the plough layer (~30 cm). The soil organic carbon (SOC), total nitrogen and C:N of surface soil layers increased under the rotary-till (0–20 cm) and no-till (0–10 cm), which were higher in 0–30 cm soil layers for residue incorporation. For both years, root characteristics of root diameter (RAD) and root surface area density (RSD), biomass indexes of root biomass (RB), shoot biomass (SB) and root-shoot ratio (R:S) were increased under these short-term treatments. Although there were positive relationships between soil water content (SWC), C:N, RAD, RSD, RB, SB, R:S and yield, structural equation modeling showed maize yield was directly controlled by R:S. These findings will have important implications for improving the current cropping system (i.e., plow-till with residue removed) in this area.

## Introduction

The brown soil region (6.8 Mha) accounts for approximately 50% of the total area (14.8 Mha) of Liaoning Province in Northeast China. Accordingly, it provides up to 60% of the total spring maize yield (11.7 Mt) of this region^1^. Using a grain-straw ratio of 2 for maize^2^, it produces approximately 23.4 Mt crop residue every year. The remaining residue always impedes the maize sowing of the next year unless local farmers remove or burn it^3^. Plowing is essential before planting because the bare top soil is often hardened during the long winter fallow period. However, years of plow-till increased the compactness and thickness of the plow layer, which might inhibit the growth of crop roots^4^. Furthermore, removing or burning residue would cause the decline of soil organic carbon (SOC) and other environmental problems (such as fire disaster and haze)^3,5,6^. In recent years, the yield instability of spring maize becomes increasingly pronounced under the traditional cropping system (plow-till with residue removed) in this region^7^.

Tillage and residue treatments are important factors affecting crop root growth. These treatments can be reflected in root characteristics of root diameter (RAD), root-length density (RLD) and root surface area density (RSD)^8,9^. Generally, short-term plow-till promotes root growth through the disturbance and inversion of soil^10,11^. However, long-term plow-till would restrict root penetration due to the formation of thick plough layer^12,13^. RAD and RSD would increase when plow-till turns to reduced tillage treatments of rotary-till and no-till^14,15^. Nevertheless, a few studies reports that RLD decreased in the no-till treatments^16,17^. Compared to residue removal, residue incorporation can increase the RAD, RLD and RSD^14,18^. This is mainly because residue decomposition is always beneficial for improving the content of SOC and soil total nitrogen^19^. Some studies find that the interaction of tillage and residue treatments can also promote crop root growth^20,21^.

Tillage and residue treatments also affect crop yield by regulating crop biomass indexes of root biomass (RB), shoot biomass (SB) and root-shoot ratio (R:S). Similarly, short-term plow-till can increase RB, SB and yield^22,23^. Long-term plow-till always restricts root growth and R:S^24^, which could cause massive maize lodging^13,25^. Compared to plow-till, rotary-till and no-till can improve RB and SB, regulate R:S and increase yield^26,27^. Residue incorporation can also increase crop biomass and yield due to the improvement of soil buffer capacity^28,29^. Furthermore, the interaction of tillage and residue treatments can obtain higher crop biomass and yield^30,31^.

Optimizing agricultural management can enhance and stabilize crop yield^12,21^. A number of previous studies have investigated tillage and residue practices on crop growth and yield in this region^7,22,32^. However, the mechanisms of maize yield under short-term reduced tillage and residue incorporation are little known^8,12,33^. In 2014 and 2015, three tillage (plow-till, rotary-till and no-till) and two residue (residue removal and residue incorporation) treatments were arranged in a split-plot experiment. The objectives of this study were to (1) identify the influences of reduced tillage and residue treatments on soil physical and chemical properties, (2) investigate the root characteristics and biomass indexes of spring maize under short-term reduced tillage and residue treatments, (3) explore the effects of short-term reduced tillage and residue incorporation on maize growth.

## Results

### Seasonal variations in precipitation and temperature

**Figure 1:**
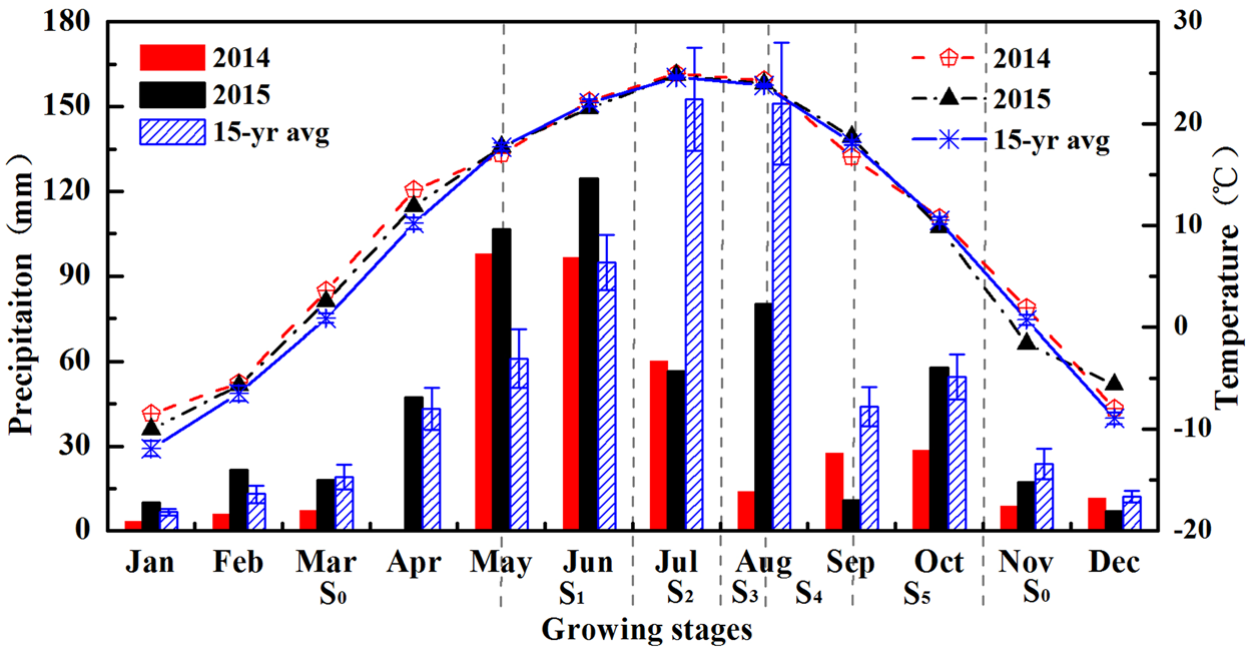
Monthly precipitation and mean temperature in 2014 and 2015. S_0_: Fallowing stage; S_1_: Seeding stage; S_2_: Jointing stage; S_3_: Silking stage; S_4_: Grain-filling stage; S_5_: Maturity stage.

Total precipitation was 362.9 mm in 2014 and 558.3 mm in 2015 (Fig. 1). The annual precipitation of 15-year (2001–2015) was 678.3 mm. During the spring maize growing season, precipitation was 41.3% (2014) and 24.8% (2015) lower than the 15-year average. Because the precipitation anomaly percentages (i.e. 41.3% in 2014 and 24.8% in 2015) exceeded 15%^34^, both study years were subjected to drought. Marked differences in air temperature were recorded over the two study years, which was 9.4 °C in 2014 and 9.0 °C in 2015. The average annual mean temperature was 8.5 °C (2001–2015). During the spring maize growing season, air temperature was 0.1 °C higher (2014) and 0.4 °C lower (2015) than the 15-year average.

### Seasonal variations in soil physical and chemical properties

From the silking (S_3_) to maturity (S_5_) stages, tillage and residue treatments had similar influences on the physical properties of 0–40 cm soil layers in 2014 and 2015 (Tables 1, S1 and S2). For illustrative purposes, only the soil physical properties at the maturity (S_5_) stage are presented as a reference (data of the other two stages are list in Tables S1 and S2, same as following next). As shown in Table 1, both of the tillage and residue treatments had significant individual effects on soil bulk density and soil water content (SWC) in 2014 and 2015. However, these treatments had no significant effects on soil compaction. Compared to plow-till, rotary-till and no-till decreased the soil bulk density and compaction below 30 cm (plough layer). Moreover, rotary-till decreased the soil bulk density and compaction in 0–10 cm. Both rotary-till and no-till increased the SWC in 0–40 cm. Under the residue incorporation treatments, soil bulk density and compaction were decreased, but the SWC were improved in 0–40 cm soil layers.

**Table 1:** Soil bulk density, soil compaction and soil water content influenced by tillage and residue treatments at the maturity stage of spring maize.

Depth (cm)	Treat-ment	Soil bulk density (g cm^−3^)				Soil compaction (cm cm^−3^)				Soil water content (%)			
		2014		2015		2014		2015		2014		2015	
		RR	RI	RR	RI	RR	RI	RR	RI	RR	RI	RR	RI
0–10	PT	1.49 ± 0.01a	1.47 ± 0.04 aA	1.50 ± 0.02a	1.45 ± 0.02a	313.9 ± 21.93b	267.0 ± 21.45b	345.0 ± 9.66ab	286.0 ± 3.90b	9.6 ± 0.91c	11.1 ± 0.06b	9.6 ± 0.05b	10.7 ± 0.98cA
	RT	1.45 ± 0.04b	1.40 ± 0.02b	1.43 ± 0.03b	1.39 ± 0.04b	228.0 ± 17.71c	199.5 ± 1.90cA	227.1 ± 12.33b	198.6 ± 21.58cA	11.6 ± 1.01b	13.4 ± 0.83a	9.7 ± 0.82b	11.5 ± 0.16b
	NT	1.51 ± 0.05a	1.48 ± 0.03 aA	1.52 ± 0.01a	1.50 ± 0.01 aA	368.0 ± 23.2a	328.9 ± 12.97a	379.5 ± 21.53	335.5 ± 8.98a	12.1 ± 1.18a	13.8 ± 0.19 aA	10.2 ± 0.37a	13.0 ± 0.21a
10–20	PT	1.44 ± 0.04b	1.43 ± 0.05bA	1.48 ± 0.04b	1.45 ± 0.04b	409.5 ± 9.67b	380.0 ± 2.45b	447.9 ± 22.2b	376.0 ± 22.20bA	10.1 ± 1.11b	11.0 ± 0.26b	10.2 ± 0.28b	11.1 ± 0.47bA
	RT	1.47 ± 0.01b	1.45 ± 0.02bA	1.50 ± 0.01ab	1.48 ± 0.05abA	421.4 ± 13.34b	398.5 ± 5.72bA	414.5 ± 21.58b	371.5 ± 9.23bA	11.3 ± 0.72a	12.9 ± 0.96 aA	10.5 ± 1.03ab	11.4 ± 0.88bA
	NT	1.54 ± 0.01a	1.52 ± 0.04 aA	1.55 ± 0.05a	1.53 ± 0.04 aA	558.9 ± 7.32a	493.9 ± 10.89a	560.1 ± 7.36a	512.6 ± 17.39 aA	10.9 ± 0.78a	13.5 ± 0.23a	10.8 ± 0.11a	12.5 ± 0.19a
20–30	PT	1.52 ± 0.01b	1.42 ± 0.01b	1.53 ± 0.04b	1.44 ± 0.03b	432.2 ± 10.56c	351.5 ± 4.03c	467.5 ± 7.49b	360.1 ± 18.40c	8.7 ± 0.68b	10.1 ± 0.39b	11.0 ± 0.36b	11.5 ± 0.96bA
	RT	1.56 ± 0.01a	1.54 ± 0.05 aA	1.56 ± 0.01a	1.53 ± 0.04 aA	534.0 ± 8.00b	523.0 ± 15.60bA	610.0 ± 24.28a	580.0 ± 11.64b	10.7 ± 0.72a	11.5 ± 0.02a	11.1 ± 0.01ab	12.0 ± 1.14b
	NT	1.57 ± 0.04a	1.56 ± 0.03 aA	1.59 ± 0.04a	1.57 ± 0.01 aA	598.0 ± 14.65a	587.0 ± 12.04 aA	631.0 ± 19.5a	611.0 ± 24.93 aA	11.0 ± 1.13a	12.5 ± 0.59a	11.5 ± 1.02a	12.7 ± 0.99 aA
30–40	PT	1.62 ± 0.05a	1.60 ± 0.06 aA	1.63 ± 0.04a	1.61 ± 0.04 aA	856.4 ± 17.76a	813.6 ± 24.49 aA	879.6 ± 13.8a	846.0 ± 7.93 aA	9.6 ± 0.49c	9.9 ± 0.41bA	10.7 ± 0.39b	11.1 ± 0.95cA
	RT	1.59 ± 0.02a	1.58 ± 0.06 aA	1.60 ± 0.05a	1.57 ± 0.02 aA	715.9 ± 22.07b	658.2 ± 6.02b	785.6 ± 9.31b	738.1 ± 16.18b	10.5 ± 0.66b	11.0 ± 1.14bA	11.9 ± 0.31ab	12.2 ± 0.45bA
	NT	1.59 ± 0.02a	1.59 ± 0.03 aA	1.61 ± 0.03a	1.60 ± 0.02 aA	601.0 ± 11.07c	591.0 ± 15.77cA	621.0 ± 2.21c	607.0 ± 22.77cA	11.1 ± 0.83a	12.0 ± 0.32 aA	12.3 ± 0.43a	12.9 ± 0.64 aA
Analysis of variance													
T		**		***		ns		ns		***		***	
R		*		*		ns		ns		***		*	
T*R		ns		ns		ns		ns		ns		ns	

PT, RT and NT indicate plow-till, rotary-till and no-till, respectively. RR and RI indicate residue removal and residue incorporation, respectively. T and gR indicate tillage and residue treatments, respectively. Values are expressed as the mean ± standard error. Different lowercase letters on mean values indicate significant differences at *P* < 0.05. Differences are significant at *P* < 0.05 between residue removal and residue incorporation under different tillage treatments except for figures marked A. *P < 0.05; **P < 0.01; ***P < 0.001; ns, not significant.

From the silking (S_3_) to maturity (S_5_) stages, tillage and residue treatments also had similar influences on the chemical properties of 0–30 cm soil layers in 2014 and 2015 (Tables 2, S3 and S4). Meanwhile, the influences on soil chemical properties of SOC, total nitrogen and C:N ratio (C:N) became more obvious during the late growing season. Tillage treatments had no significant effects on SOC, total nitrogen and C:N. But residue treatments had significant effects on the soil chemical properties, especially the soil layers incorporated residue. For the residue treatment, residue incorporation increased SOC, total nitrogen and C:N in 0–30 cm soil layers.

**Table 2:** Soil organic carbon, total nitrogen and C:N ratio influenced by tillage and residue treatments at the maturity stage of spring maize.

Depth (cm)	Treat-ment	Soil organic carbon (g kg^−1^)				Total nitrogen (g kg^−1^)				C:N ratio			
		2014		2015		2014		2015		2014		2015	
		RR	RI	RR	RI	RR	RI	RR	RI	RR	RI	RR	RI
0–10	PT	13.2 ± 0.07c	13.9 ± 0.02c	13.7 ± 0.06c	14.1 ± 0.07c	0.98 ± 0.01b	1.03 ± 0.01b	1.00 ± 0.04c	1.04 ± 0.03bA	13.42 ± 0.01c	13.45 ± 0.05c	13.45 ± 0.05b	13.48 ± 0.04c
	RT	13.9 ± 0.05b	14.6 ± 0.06b	14.2 ± 0.04b	14.7 ± 0.04b	1.03 ± 0.01a	1.08 ± 0.04a	1.05 ± 0.02b	1.08 ± 0.01a	13.47 ± 0.02b	13.55 ± 0.02b	13.43 ± 0.05b	13.56 ± 0.05b
	NT	14.4 ± 0.07a	15.1 ± 0.05a	14.9 ± 0.03a	15.3 ± 0.01a	1.06 ± 0.01a	1.11 ± 0.04a	1.09 ± 0.01a	1.11 ± 0.02a	13.61 ± 0.06a	13.64 ± 0.04a	13.67 ± 0.03a	13.70 ± 0.01a
10–20	PT	13.9 ± 0.04b	14.1 ± 0.02bA	14.4 ± 0.03b	14.8 ± 0.05b	1.03 ± 0.02a	1.04 ± 0.01bA	1.06 ± 0.02a	1.09 ± 0.04a	13.54 ± 0.02b	13.55 ± 0.06bA	13.57 ± 0.02b	13.59 ± 0.05bA
	RT	14.3 ± 0.03a	15.0 ± 0.05a	14.6 ± 0.03a	15.3 ± 0.02a	1.05 ± 0.04a	1.10 ± 0.03a	1.07 ± 0.02a	1.12 ± 0.01a	13.57 ± 0.05a	13.63 ± 0.03a	13.61 ± 0.06a	13.64 ± 0.01a
	NT	13.6 ± 0.01c	13.8 ± 0.03cA	13.7 ± 0.01c	14.0 ± 0.08cA	1.00 ± 0.04b	1.02 ± 0.02bA	1.01 ± 0.01b	1.03 ± 0.01bA	13.50 ± 0.03c	13.54 ± 0.01bA	13.52 ± 0.01c	13.56 ± 0.05c
20–30	PT	14.4 ± 0.04a	15.3 ± 0.03a	14.5 ± 0.03a	15.4 ± 0.02a	1.06 ± 0.01a	1.13 ± 0.02a	1.07 ± 0.03a	1.13 ± 0.04a	13.58 ± 0.02a	13.62 ± 0.03a	13.61 ± 0.02a	13.67 ± 0.01a
	RT	13.5 ± 0.06b	13.9 ± 0.04bA	13.6 ± 0.06b	13.9 ± 0.07bA	1.00 ± 0.03b	1.03 ± 0.01b	1.01 ± 0.02b	1.02 ± 0.04bA	13.48 ± 0.03b	13.51 ± 0.02cA	13.53 ± 0.04c	13.61 ± 0.02b
	NT	12.9 ± 0.07c	13.2 ± 0.01cA	13.0 ± 0.05c	13.3 ± 0.01cA	0.95 ± 0.01c	0.97 ± 0.01cA	0.96 ± 0.04c	0.97 ± 0.02cA	13.57 ± 0.03a	13.58 ± 0.01bA	13.58 ± 0.01b	13.60 ± 0.02bA
Analysis of variance													
T		ns		ns		ns		*		ns		ns	
R		**		*		**		*		*		*	
T*R		ns		ns		ns		ns		ns		ns	

PT, RT and NT indicate plow-till, rotary-till and no-till, respectively. RR and RI indicate residue removal and residue incorporation, respectively. T and R indicate tillage and residue treatments, respectively. Values are expressed as the mean ± standard error. Different lowercase letters on mean values indicate significant differences at *P* < 0.05. Differences are significant at *P* < 0.05 between residue removal and residue incorporation under different tillage treatments except for figures marked A. *P < 0.05; **P < 0.01; ***P < 0.001; ns, not significant.

### Seasonal variations in root characteristics and biomass indexes

From the silking (S_3_) to maturity (S_5_) stages, tillage and residue treatments had similar influence trends on root diameter (RAD), root-length density (RLD) and root surface area density (RSD) in 2014 and 2015 (Tables 3, S5 and S6). Treatments of tillage and residue had significant individual effects on RAD, RLD and RSD. Both the RAD and RSD were improved under rotary-till and no-till, while the RLD was decreased compared with plow-till. It should be noted that reduced tillage commonly had a more effective influence on root characteristics during later growing periods. Compared to residue removal, residue incorporation improved the RAD, RLD and RSD of spring maize.

**Table 3:** Root diameter, root-length density and root surface area density influenced by tillage and residue treatments at the maturity stage of spring maize.

Treatment	Root diameter (mm)				Root-length density (cm cm^−3^)				Root surface area density (cm^2^ cm^−3^)			
	2014		2015		2014		2015		2014		2015	
	RR	RI	RR	RI	RR	RI	RR	RI	RR	RI	RR	RI
PT	3.31 ± 0.27b	3.75 ± 0.31b	3.42 ± 0.27b	3.68 ± 0.31b	4.10 ± 0.34a	3.55 ± 0.30a	4.13 ± 0.37a	4.55 ± 0.42a	0.81 ± 0.24b	0.98 ± 0.28b	1.00 ± 0.26b	1.09 ± 0.30c
RT	3.38 ± 0.31b	3.80 ± 0.37b	3.45 ± 0.31b	3.74 ± 0.37b	3.82 ± 0.33b	3.27 ± 0.28b	3.91 ± 0.34b	4.35 ± 0.38b	0.86 ± 0.24a	1.00 ± 0.28a	1.06 ± 0.25a	1.11 ± 0.28b
NT	3.67 ± 0.43a	4.06 ± 0.49a	3.73 ± 0.45a	4.10 ± 0.49a	3.66 ± 0.37b	3.20 ± 0.27b	3.74 ± 0.37b	4.14 ± 0.43b	0.90 ± 0.24a	1.02 ± 0.32a	1.10 ± 0.22a	1.16 ± 0.31a
Analysis of variance												
T	**		**		**		**		*		***	
R	***		**		***		***		***		***	
T*R	ns		ns		ns		ns		ns		**	

PT, RT and NT indicate plow-till, rotary-till and no-till, respectively. RR and RI indicate residue removal and residue incorporation, respectively. T and R indicate tillage and residue treatments, respectively. Values are expressed as the mean ± standard error. Different lowercase letters on mean values indicate significant differences at *P* < 0.05. Differences are significant at *P* < 0.05 between residue removal and residue incorporation under different tillage treatments except for figures marked A. *P < 0.05; **P < 0.01; ***P < 0.001; ns, not significant.

Similar to the root characteristics, influence of different treatments on biomass indexes of spring maize became more obvious during the late growing season (Tables 4, S7 and S8). Tillage treatments only had significant effects on root biomass (RB) and root-shoot ratio (R:S) of spring maize. Residue treatments had significant effects on RB, shoot biomass (SB) and R:S. Nevertheless, the effects of residue treatments on SB were not significant at the S_3_ and S_4_ stages. Compared to plow-till, rotary-till and no-till increased the RB, SB and R:S. With respect to the residue treatments, residue incorporation also increased the RB, SB and R:S.

**Table 4:** Root biomass, shoot biomass and root-shoot ratio influenced by tillage and residue treatments at the maturity stage of spring maize.

Treat-ment	Root biomass (g plant^−1^)				Shoot biomass (g plant^−1^)				Root-shoot ratio			
	2014		2015		2014		2015		2014		2015	
	RR	RI	RR	RI	RR	RI	RR	RI	RR	RI	RR	RI
PT	14.0 ± 0.44b	17.5 ± 1.93b	14.0 ± 0.40b	19.2 ± 3.84b	336.8 ± 37.05b	377.0 ± 9.87b	335.1 ± 2.35b	364.0 ± 11.40b	0.042 ± 0.005b	0.046 ± 0.006b	0.042 ± 0.002b	0.046 ± 0.014b
RT	15.5 ± 0.92b	18.6 ± 1.94b	15.5 ± 0.35b	19.3 ± 3.86b	339.2 ± 37.31b	378.2 ± 11.91b	346.7 ± 7.63b	380.0 ± 0.43b	0.046 ± 0.005a	0.049 ± 0.004a	0.045 ± 0.001a	0.051 ± 0.010a
NT	18.0 ± 0.38a	20.0 ± 0.63a	18.0 ± 0.40a	20.0 ± 0.52a	356.1 ± 39.18a	390.7 ± 13.88a	374.0 ± 1.00a	398.0 ± 5.29a	0.051 ± 0.007a	0.051 ± 0.003a	0.048 ± 0.001a	0.050 ± 0.002a
Analysis of variance												
T	**		**		ns		***		*		*	
R	***		**		*		***		*		*	
T*R	ns		ns		ns		ns		ns		ns	

PT, RT and NT indicate plow-till, rotary-till and no-till, respectively. RR and RI indicate residue removal and residue incorporation, respectively. T and R indicate tillage and residue treatments, respectively. Values are expressed as the mean ± standard error. Different lowercase letters on mean values indicate significant differences at *P* < 0.05. Differences are significant at *P* < 0.05 between residue removal and residue incorporation under different tillage treatments except for figures marked A. *P < 0.05; **P < 0.01; ***P < 0.001; ns, not significant.

### Spring maize yield analysis

Table 5 lists the influence of tillage and residue treatments on spring maize yield in 2014 and 2015. Tillage and residue treatments significantly influenced the yield of spring maize. However, the interaction effect was not significant. Yield was higher under rotary-till (15.9%) and no-till (30.7%) treatments, the difference between plow-till and no-till was significant. Compared to residue removal, residue removal also significantly increased the yield of spring maize (7.2%).

**Table 5:** Spring maize yield influenced by tillage and residue treatments in 2014 and 2015.

Treatment	Yield (t ha^−1^)			
	2014		2015	
	RR	RI	RR	RI
PT	9.9 ± 0.76c	10.65 ± 0.43b	8.1 ± 0.10c	8.63 ± 0.46c
RT	10.57 ± 0.51b	10.79 ± 0.41b	9.97 ± 0.53b	11.44 ± 0.61b
NT	11.05 ± 0.18a	11.7 ± 0.46a	12.23 ± 0.65a	12.98 ± 0.69a
Analysis of variance				
T	**		***	
R	*		**	
T*R	ns		ns	

PT, RT and NT indicate plow-till, rotary-till and no-till, respectively. RR and RI indicate residue removal and residue incorporation, respectively. T and R indicate tillage and residue treatments, respectively. Values are expressed as the mean ± standard error. Different lowercase letters on mean values indicate significant differences at *P* < 0.05. Differences are significant at *P* < 0.05 between residue removal and residue incorporation under different tillage treatments except for figures marked A. *P < 0.05; **P < 0.01; ***P < 0.001; ns, not significant.

## Discussion

This study investigated the effects of tillage and residue treatments on soil properties and crop growth and their relation to grain yield of spring maize in Northeast China. Our results indicated that short-term reduced tillage (rotary-till and no-till) and residue incorporation promoted soil physical-chemical properties, maize growth and grain yield.

### Effects of soil physical and chemical properties on yield

**Figure 2:**
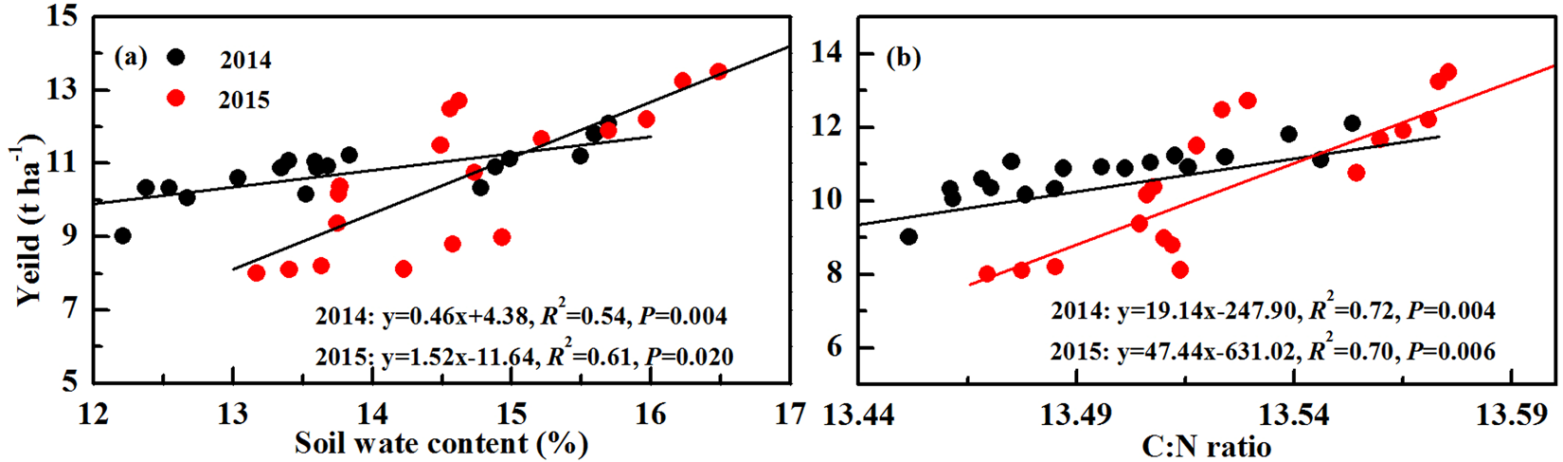
Linear relationship of soil water content (**a**) and C:N ratio (**b**) on yield of spring maize in 2014 and 2015. Data was obtained from the silking, grain-filling and maturity stages with three replications for each stage.

Our results indicated that short-term reduced tillage (rotary-till and no-till) decreased the soil bulk density and compaction below the plough layer (~30 cm). Meanwhile, they had higher SWC in the 0–40 cm soil layer than that of plow-till (Table 1). The greater soil bulk density and compaction of plow-till might be due to the thick plough layer caused by long-term excessive tillage^19,35^. Less porosity leaded by reduced tillage might be an important reason for the higher SWC of rotary-till and no-till^33,36^. Apparently, residue decomposition could help to decrease the soil bulk density and compaction due to the increased soil stable aggregate^37,38^. Residue incorporation also enhanced the capacity of soil water retention, which in turn increased the SWC^39^. As shown in Fig. 2a, there was a positive relationship between SWC and the yield of spring maize in 2014 (y = 0.46x + 4.38, *R*² = 0.54, *P* = 0.004) and 2015 (y = 1.52x − 11.64, *R*² = 0.61, *P* = 0.020). But the soil bulk density and compaction were not significantly related with spring maize yield (*P* > 0.05). This illustrated that SWC was a major physical property influencing the grain yield of spring maize^33^.

Short-term reduced tillage increased the SOC, total nitrogen and C:N of surface soil layers for rotary-till (0–20 cm) and no-till (0–10 cm) (Table 2). Soil fertility in surface soil layers increased and accumulated under reduced tillage possibly due to the minimum soil disturbance^19,35^. Frequent plow-till would cause more soil disturbance, which accelerated the mineralization of soil organic matters^40,41^. Limousin and Tessier^41^ and Dai *et al*.^19^ found that SOC and total nitrogen were accumulated at topsoil in no-till with an obvious centration gradient from the surface to subsoil. Residue only increased the soil chemical properties of tillage layers (i.e. 0–30 cm for plow-till, 0–20 cm for rotary-till and 0–10 cm for no-till). This mainly because tillage treatments increased the contact between residue and soil microbes, which promoted the decomposition process and increased soil fertility^42^. As shown in Fig. 2b, only C:N and maize yield were positively related in 2014 (y = 19.14x − 247.90, *R*² = 0.72, *P* = 0.004) and 2015 (y = 47.44x − 631.02, *R*² = 0.70, *P* = 0.006), indicating it was the major chemical property influencing the grain yield of spring maize^43^. Zhang *et al*.^12^ also indicated that rotary-till and no-till could obtain a good harvest through higher C:N for greater capacity of the soil to store and recycle nutrients and energy.

### Effects of root characteristics and biomass indexes on yield

**Figure 3:**
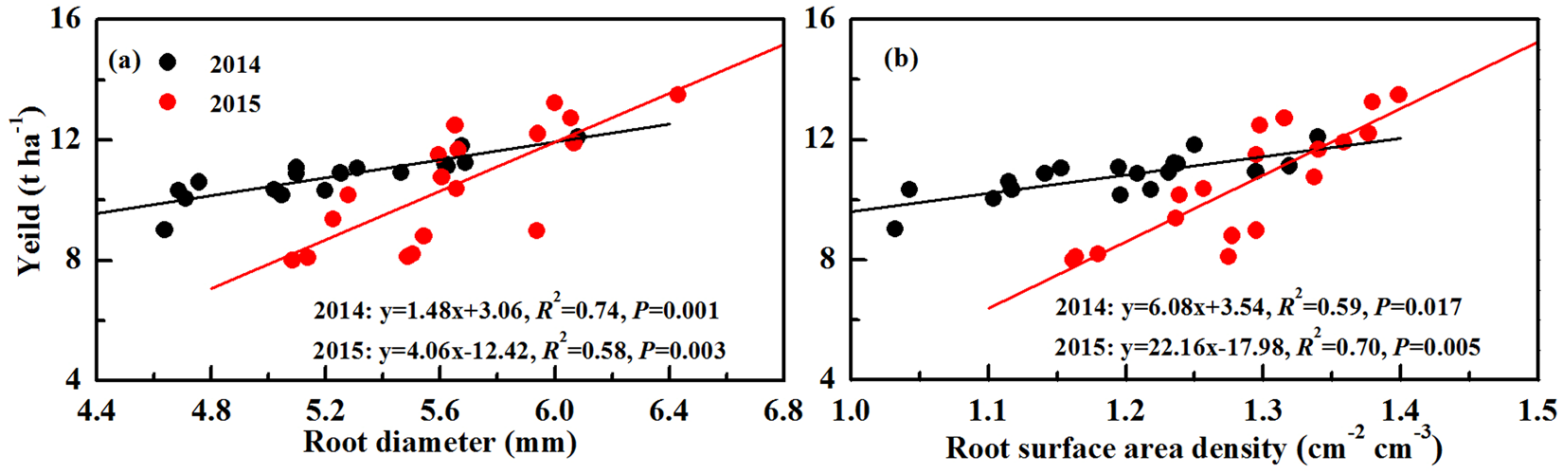
Linear relationship of root diameter (**a**) and root surface area density (**b**) on yield of spring maize in 2014 and 2015. Data was obtained from the silking, grain-filling and maturity stages with three replications for each stage.

We found that short-term reduced tillage (rotary-till and no-till) had an increasing effect on root diameter (RAD) and root surface area density (RSD), while a decreasing effect of root-length density (RLD) (Table 3). The higher soil bulk density and compaction in 0–30 cm soil layer not only coarsen root diameter and increased RAD and RSD^44^, but also restricted root penetration and decreased RLD^16,17^. Increased SWC and SOC under reduced tillage (Tables 1 and 2) might be another reason for RAD and RSD increasement^6,20^. As regards to residue treatment, residue increased all the root characteristics of spring maize (Table 3). Crop residue decreased soil compaction and increased SWC (Table 1), which was conducive to root distribution and growth^18,45^. Crop residue also incorporated into the soil as a source of SOC and total nitrogen, which could be another important reason for increasing RAD, RLD and RSD^46,47^. There was a positive relationship between RAD and yield both in 2014 (y = 1.48x + 3.06, *R* ^2^ = 0.74, *P* = 0.001) and 2015 (y = 4.06x−12.42, *R* ^2^ = 0.58, *P* = 0.003) (Fig. 3). Similarly, RSD was also positively correlated with yield in 2014 (y = 6.08x + 3.54, *R* ^2^ = 0.59, *P* = 0.017) and 2015 (y = 22.16x − 17.98, *R* ^2^ = 0.70, *P* = 0.005). These suggested that RAD and RSD might play important roles in yield. Similarly, Guan *et al*.^23^ reported that higher RSD and active root system presented a close relation to higher yield, because of the efficient substance-transfer mechanism from crop roots to shoots.

**Figure 4:**
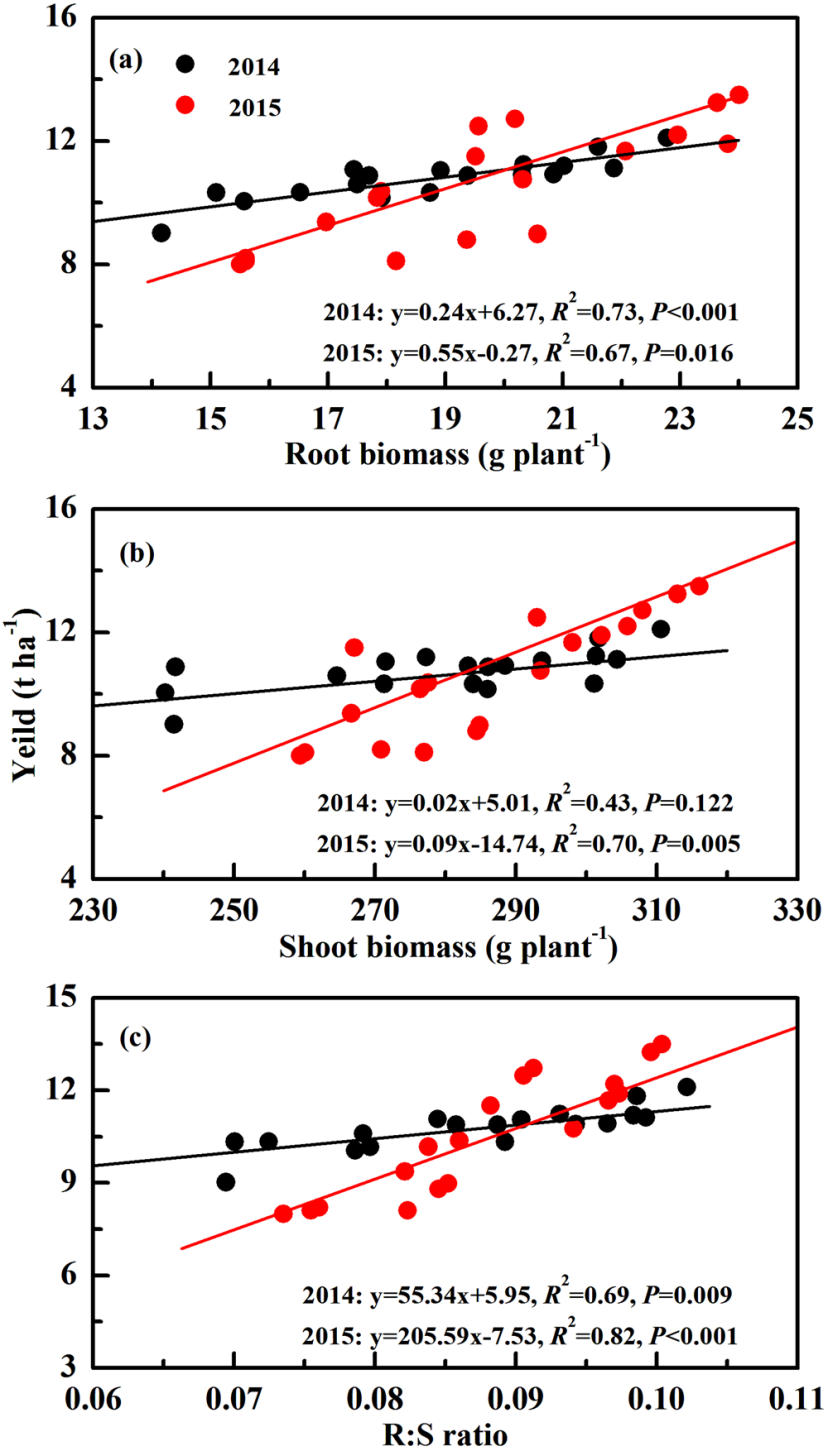
Linear relationship of root biomass (**a**), shoot biomass (**b**) and R:S ratio (**c**) on yield of spring maize in 2014 and 2015. Data was obtained from the silking, grain-filling and maturity stages with three replications for each stage.

Short-term reduced tillage promoted root biomass (RB), shoot biomass (SB) and root-shoot ratio (R:S) (Table 4). Higher porosity and lower compaction in subsoil layer provided a suitable (less restricted) soil physical environment for root growth and distribution^36^. Moreover, the greater RAD and RSD under reduced tillage (Table 3) promoted the absorption of water and nutrients, which also promoted crop growth and increased RB and SB^23,48^. Passioura^49^ suggested that there was an optimum R:S for a given water supply. Higher R:S under conservation tillage was of a vital importance to support crop structure and enhance grain yield, especially in droughty conditions^48^. Crop residue facilitated soil water infiltration and provided a buffer for drought episodes, which was beneficial for promoting crop root and shoot biomass^50–52^. Moreover, residue incorporation increased SOC, total nitrogen and C:N (Table 2), which could provide nutritional support for crop biomass accumulation^53^. The relationship between RB and maize yield in 2014 (y = 0.24x + 6.27, *R* ^2^ = 0.73, *P* < 0.001) and 2015 (y = 0.55x − 0.27, *R* ^2^ = 0.67, *P* = 0.016) were shown in Fig. 4. Similarly, R:S also positively correlated with yield in 2014 (y = 55.348x + 5.95, *R* ^2^ = 0.69, *P* = 0.009) and 2015 (y = 205.59x − 7.53, *R* ^2^ = 0.82, *P* < 0.001). However, SB had a significant relationship with yield only in 2015 (y = 0.09x − 14.74, *R* ^2^ = 0.70, *P* = 0.005). The more obvious relationship between RB, R:S and yield may be because short-term tillage and residue treatments had greater effects on root systems than did plant shoots^24^.

### Potential mechanism of yield response to tillage and residue treatments

In order to gain a mechanistic understanding of how tillage and residue affected spring maize yield, the structural equation modeling (SEM) was used in this study. SWC, C:N, root diameter (RAD), root surface area density (RSD), shoot biomass (SB) and root-shoot ratio (R:S) passed the test of regression analysis, and were used for the SEM. This model provided an excellent fit to our data based on the indexes of model fit (χ^2^ = 15.187, df = 23, *P* = 0.372; χ^2^ = 12.783, df = 23, *P* = 0.496). The variables revealed that the predictors explained 61% and 63% of maize yield in 2014 and 2015, respectively.

**Figure 5:**
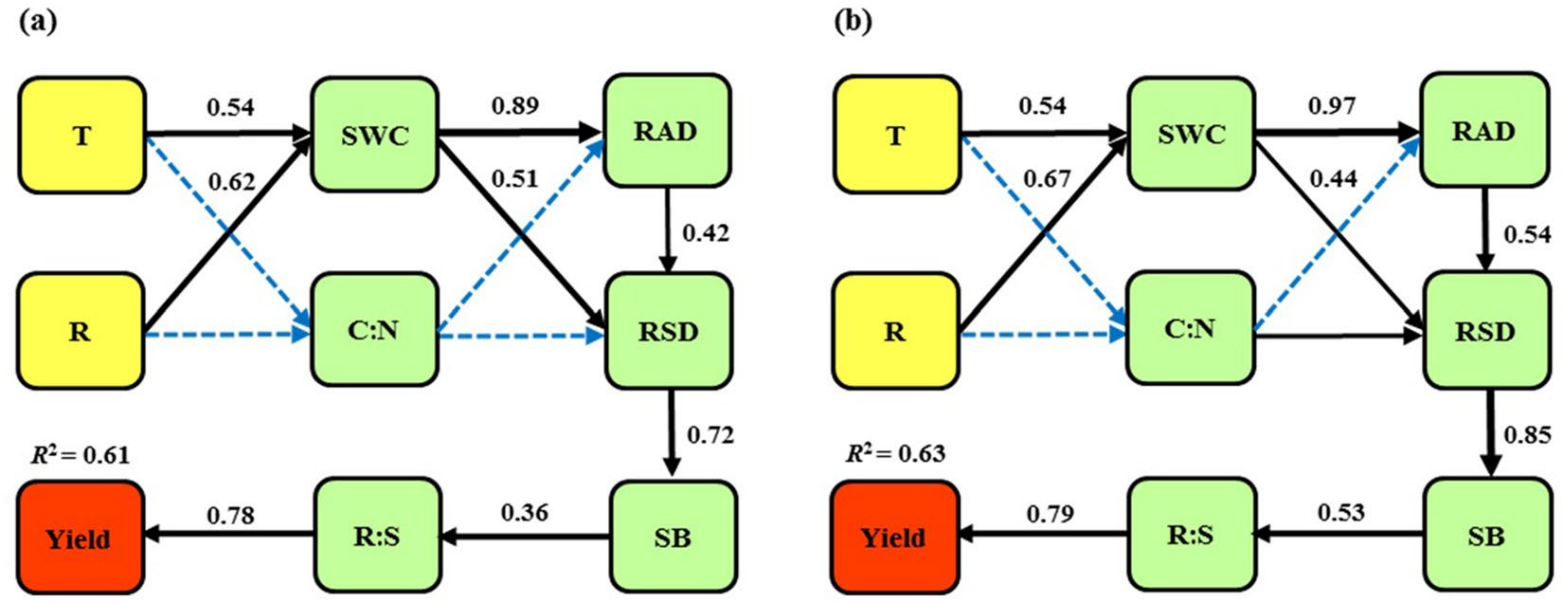
Structural equation model relating tillage and residue treatments to yield of spring maize in 2014 (**a**) and 2015 (**b**). T and R in the boxes represent tillage and residue treatments, respectively. Boxes represent variables past the Pearson test and Regression analysis. Arrows show the direct effects of one variable on the others. Values next to the arrows are standardized path coefficients. Solid and dashed lines indicate significance (*P* < 0.05) and non-significance (*P* > 0.05).

Based on the model results, we found that tillage and residue treatments affected yield indirectly through SWC, while the effect of C:N on yield was not significant (Fig. 5). Furthermore, SWC directly regulated RAD (path coefficient = 0.89 in 2014 and 0.97 in 2015) and RSD (0.51 in 2014 and 0.44 in 2015), indicating it affected yield through root morphological characteristics. Previous studies have proved that SWC had significant effects on crop yield through stimulating root distribution and deep growth^26,45,51^. RSD, affected by RAD directly either (0.42 in 2014 or 0.54 in 2015), contributed to yield indirectly through SB. The model further demonstrated that SB was the strongest indirect factor on yield through R:S in 2014 (path coefficient = 0.36) and 2015 (path coefficient = 0.53). Plaza-Bonilla *et al*.^24^ found that greater root biomass under reduced tillage had obvious improvement to shoot growth and grain yield. Moreover, R:S, as the direct and key acting factor, made the strongest contribution to spring maize yield (0.78 in 2014 and 0.79 in 2015). This suggested that reduced tillage and residue promoted yield for the higher R:S provided more water and nutrition for crop growth and matter accumulation, especially in drought environment^48^.

Our study found that short-term reduced tillage and residue incorporation promoted spring maize yield through increasing soil physical-chemical property, root characteristics and biomass indexes in the growing season. Linear analysis showed positive relationships between yield and soil properties of SWC and C:N, root characteristics of RAD and RSD and biomass indexes of RB, SB and R:S. SEM results further suggested that reduced tillage and residue incorporation increased yield through regulating R:S directly. These findings indicated that short-term reduced tillage and residue incorporation could be a potential alternative to the traditional plow-till in the brown soil of Northeast China. However, the influence of long-term tillage and residue practices on soil properties and crop growth remains unclear. Therefore, further studies should be implemented to reveal the mechanism of long-term reduced tillage and residue incorporation on soil properties and crop growth and their contribution to grain yield in this area.

## Methods

### Experimental site

The experiment was conducted at the Experimental Station (41°82′N, 123°56′E, 43 m a.s.l.) of Shenyang Agricultural University in Liaoning province, China. This region has a sub-humid warm temperate continental climate. The mean annual temperature is 7.9 °C, the mean annual precipitation is 714 mm and more than 65% of the precipitation occurs during the rainy season (June to September). The average annual frost-free period is 155–180 days (2001–2015). The soil texture is brown soil. According to the measurement at the beginning of this study, the content of SOC, total nitrogen and total phosphorus was 14.6 g kg^−1^, 1.05 g kg^−1^ and 0.85 g kg^−1^, respectively. The main crop in this region is spring maize (*Zea mays* L.). The crop-planting pattern in this area is one harvest per year. The traditional tillage measure is plow-till with crop residue removed. Experimental plots had no irrigation for all treatments and all the crop water requirements were provided by natural precipitation.

### Experimental design and management practices

The experiment was a split-plot design with three replicates. At the main plot level, the three tillage treatments were plow-till, rotary-till and no-till. At the subplot level, the two residue treatments were residue removal and residue incorporation. The main plot size was 12 m × 8 m and was split into two 6 m × 8 m subplots.

In the residue removal subplots, maize residues were removed from the field. Plow-till inverted the soil to a depth of 25 cm with a plow (1L-525, Baoding Agriculture Machinery Co., Ltd.). Accordingly, the rotary-pill smashed the soil at a depth of 0–15 cm with a rotary tiller (1GKN-240, Tianfeng Machinery Co. Ltd.). Under the no-till treatment, the only disturbances to the soil were planting and fertilizing.

In the residue incorporation subplots, residues were fully returned at 6,000 kg ha^−1^ (dry weight). First, residues were chopped into approximately 3–5 cm pieces with a chopper (9ZP-1.2, Nongliang Agriculture Machinery Co., Ltd.). The chopped residues were flattened on the soil surface. Plow-till and rotary-till were conducted as described above. The residues in these plots were buried to 25 cm depth (plow-till) or incorporated into 0–15 cm soil layers (rotary-till). The soil surface was covered with nylon nets (3 cm × 3 cm mesh) to prevent the wind from blowing residues away from the no-till plot.

Spring maize (Zhengdan 958) was planted on May 10, 2014 and May 15, 2015. Maize was harvested on September 28, 2014 and September 30, 2015. Crops were planted at 67,500 plants ha^−1^ in 60 cm rows. Total nitrogen and total carbon content of the residues were 8.63 g kg^−1^ and 440.84 g kg^−1^, respectively. Chemical fertilizer was applied according to the local recommendation, which included 104.4 kg ha^−1^ of N, 32.8 kg ha^−1^ of P and 108 kg ha^−1^ of K. No fertilizer was top dressed during the growth period.

### Soil sample and analysis

Soil samples were collected according to a systematic sampling design according the S-shape transects at the seeding (S_1_, 17 days after seeding (DAS) in 2014 and 18 DAS in 2015), jointing (S_2_, 51 DAS in 2014 and 54 DAS in 2015), silking (S_3_, 75 DAS in 2014 and 76 DAS in 2015), grain-filling (S_4_, 102 DAS in 2014 and 104 DAS in 2015) and maturity (S_5_, 133 DAS in 2014 and 135 DAS in 2015) stages using a manual soil sampler (5 cm diameter). The S_1_ and S_2_ stages were ignored in measuring sample analysis. Five soil samples were collected from every plot at three depths (0–10, 10–20, 20–30 cm) for SOC and total nitrogen analyses. The samples were composited and mixed to form a single sample per plot for each depth. Visible plant residues and stones were removed. Soil were passed through a 2-mm sieve and stored after air-drying. The SOC and total nitrogen were determined using a FlashEA 1112 elemental analyzer (Thermo Finnigan, Italy). The C:N was computed by dividing the SOC concentration with that of total nitrogen for same depth^54^.

Soil bulk density and SWC were measured using the cutting-ring method^33^. The stainless cutting-ring was 5 cm in diameter and 5 cm in height. Five points were selected for each layer. Compaction was measured with the SC900 digital compactness instrument (Spectrum Technologies, Inc., Plainfield, IL, USA). Soil bulk density, SWC and compaction were measured at four depths of 0–10, 10–20, 20–30 and 30–40 cm at the S_3_, S_4_, and S_5_ stages.

### Root sample and analysis

In this study, maize roots were sampled at the S_3_, S_4_ and S_5_ stages. The S_1_ and S_2_ stages were ignored, mainly due to the obvious errors in measuring small roots. Three soil cores were sampled with a soil auger at three separate locations including planting spots, intra-plant in the rows and intra-rows’ spots. Cores were obtained at 10 cm increments down to 100 cm. To acquire maize roots, the soil cores were mixed together, flushed with water and filtered through a 2 mm sieve. These roots were scanned with a scanner (Epson V700, Indonesia). RAD, root length and root surface area were directly obtained using WinRHIZO software (V5.0, Regent Instruments Inc.). The RLD and RSD were calculated indirectly based on the measurements of root length and root surface area^8^.

### Biomass and yield analyses

Three maize plants at the S_3_, S_4_ and S_5_ stages were randomly selected in each plot, and the aboveground plants were cut at the soil surface. A soil sample measuring 25 cm × 60 cm × 40 cm was taken from the soil near the sampling maize. The soil cubes were then washed with water and filtered through a 2-mm sieve. RB and SB were determined by drying the root and aboveground plant in an oven at 80 °C for 48 h. The R:S was calculated as the ratio of RB to SB.

The maize yield was determined by hand harvesting the middle six rows of each plot. The grains were separated from the air-dried cob by hand. The grain moisture content was measured with a grain moisture-measuring instrument (K.T. PM-8188-A, Japan). Maize yield was standardized to 13% moisture content.

### Statistical analysis

Analysis of variance (ANOVA) was performed to assess the effects of tillage and residue treatments on soil physical and chemical properties, root characteristics, biomass indexes and yield of spring maize using SPSS statistical software (SPSS Inc., Chicago, IL). To detect differences among tillage measures, multiple comparisons were conducted by the least significant difference (LSD). Under residue measures, mean values were compared using paired *t*-tests. Differences at *P* < 0.05 level were considered statistically significant. The relationships between soil physical and chemical properties, root characteristics, biomass indexes and yield were explored using linear regression. The cause-effect relationships between soil physical and chemical properties, root characteristics, biomass indexes and spring maize yield were determined using a structural equation modeling (SEM). SEM analysis disentangled the effect into direct and indirect effects. χ^2^ and P values were used to test the validity of the model using Amos18.0 software (IBM SPSS, Amos Development Corporation, Meadville, Pennsylvania, USA).

## Electronic supplementary material

Supplementary Information
