# Supplementary material for: Short-term effects of tillage and residue on spring maize yield through regulating root-shoot ratio in Northeast China
Source: Sci Rep. 2017 Oct 17;7:13314. doi: 10.1038/s41598-017-13624-5 (PMC5645370; doi:10.1038/s41598-017-13624-5)
Supplement: Supplementary file 1 — Supplementary Information [file 41598_2017_13624_MOESM1_ESM.pdf]

Supplementary Information to ‘Short-term effects of tillage and residue on spring maize yield through regulating root-shoot ratio in Northeast China,’ by Debao You, Ping Tian, Pengxiang Sui, Wenke Zhang, Bin Yang and Hua Qi.

Supplementary Table S1. Soil bulk density, soil compaction and soil water content influenced by tillage and residue treatments at the silking stage of spring maize.

Depth (cm)	Treatment	Soil bulk density (g cm^{-3})				Soil compaction (cm cm^{-3})				Soil water content (%)			
		2014		2015		2014		2015		2014		2015	
		RR	RI	RR	RI	RR	RI	RR	RI	RR	RI	RR	RI
0-10	PT	1.34±0.03b	1.30±0.02bA	1.37±0.04b	1.29±0.05b	136.5±1.91b	120.0±24.02bA	152.3±14.79b	121.0±16.19b	12.7±0.51c	13.6±0.34bA	16.2±0.80b	18.2±0.92bA
	RT	1.32±0.04b	1.23±0.04b	1.29±0.05c	1.22±0.05c	112.1±8.47c	66.5±21.89c	114.9±14.94c	75.0±12.02c	13.7±0.55b	17.3±0.39a	17.1±1.09ab	21.7±0.43a
	NT	1.40±0.05a	1.34±0.03a	1.42±0.02a	1.35±0.04a	218.5±1.11a	154.0±16.78a	256.5±10.11a	182.6±12.17a	14.9±0.89a	17.5±0.11a	18.1±0.65a	22.9±0.67a
10-20	PT	1.37±0.05b	1.30±0.05b	1.35±0.01b	1.31±0.02bA	291.9±12.55b	240.0±21.21b	281.4±15.78b	239.0±24.76bA	14.1±0.98b	16.4±0.05b	17.0±1.17b	18.4±0.75aA
	RT	1.40±0.01b	1.32±0.01b	1.37±0.05b	1.33±0.02bA	310.2±19.21b	246.1±19.45b	272.4±9.69b	244.2±5.29bA	14.5±0.60b	19.3±0.72a	17.7±0.22a	19.1±0.45a
	NT	1.52±0.02a	1.49±0.02aA	1.52±0.03a	1.48±0.03aA	396.8±24.78a	330.0±11.07a	423.2±4.41a	379.5±17.79aA	15.9±0.12a	19.1±0.35a	18.0±0.57a	19.1±0.37aA
20-30	PT	1.40±0.05b	1.28±0.06c	1.39±0.05b	1.30±0.03c	348.9±20.21c	285.0±11.22c	362.6±5.99b	284.1±18.78c	16.5±0.65b	18.4±0.07bA	18.4±0.19b	19.8±0.60a
	RT	1.50±0.05a	1.45±0.02b	1.50±0.06a	1.46±0.05bA	429.0±22.16b	421.0±20.88bA	489.0±22.05a	458.4±16.85bA	16.8±0.87b	18.8±0.18b	19.1±0.20b	20.0±1.11bA
	NT	1.54±0.01a	1.52±0.04aA	1.55±0.04a	1.53±0.04aA	447.0±2.60a	443.0±16.49aA	499.0±2.09a	488.1±20.03aA	17.0±0.12a	20.3±0.67a	20.1±0.60a	21.4±0.18aA
30-40	PT	1.53±0.03a	1.48±0.05bA	1.52±0.05a	1.49±0.05aA	775.4±8.11a	720.7±2.35a	783.9±8.97a	754.3±4.18aA	18.4±0.64b	19.2±0.34bA	22.1±0.73b	23.1±0.70bA
	RT	1.54±0.02a	1.51±0.05abA	1.54±0.02a	1.51±0.05aA	589.5±21.38b	539.7±10.98bA	629.9±3.60b	590.5±1.01bA	19.3±0.59a	21.3±0.07a	22.5±0.64ab	24.2±0.61aA
	NT	1.55±0.01a	1.54±0.03aA	1.56±0.01a	1.54±0.03aA	477.0±24.4c	467.0±23.82cA	512.0±23.63c	496.7±6.56cA	19.6±0.90a	21.4±0.68aA	24.0±1.15a	24.6±0.59aA
Analysis of variance													
T		***		***		ns		ns		*		*	
R		*		*		ns		ns		***		**	
T*R		ns		ns		ns		ns		ns		ns	

PT, RT and NT indicate plow-till, rotary-till and no-till, respectively. RR and RI indicate residue removal and residue incorporation, respectively. T and R indicate tillage and residue treatments, respectively. Values are expressed as the mean ± standard error. Different lowercase letters on mean values indicate significant differences at $P < 0.05$. Differences are significant at $P < 0.05$ between residue removal and residue incorporation under different tillage treatments except for figures marked A. *$P < 0.05$; **$P < 0.01$; ***$P < 0.001$; ns, not significant.

Supplementary Table S2. Soil bulk density, soil compaction and soil water content influenced by tillage and residue treatments at the grain-filling stage of spring maize.

Depth (cm)	Treatment	Soil bulk density (g cm^{-3})				Soil compaction (cm cm^{-3})				Soil water content (%)			
		2014		2015		2014		2015		2014		2015	
		RR	RI	RR	RI	RR	RI	RR	RI	RR	RI	RR	RI
0-10	PT	1.40±0.04b	1.36±0.04aA	1.41±0.06a	1.35±0.03b	253.1±16.82b	221.0±16.75bA	268.8±4.39b	223.0±12.59b	13.2±0.88b	14.0±0.21bA	9.0±0.11c	9.4±0.21cA
	RT	1.38±0.06b	1.31±0.06b	1.35±0.03b	1.29±0.02b	200.5±19.34c	171.0±14.61cA	193.8±8.78c	169.1±4.44cA	13.3±1.14b	15.1±0.19aA	9.8±0.98b	11.1±0.18bA
	NT	1.43±0.02a	1.40±0.04aA	1.46±0.02a	1.42±0.01a	342.7±10.55a	257.4±1.54aA	357.7±19.27a	309.1±13.41a	13.6±0.72a	15.4±0.66a	10.8±1.07a	12.5±0.05a
10-20	PT	1.38±0.04c	1.35±0.04bA	1.39±0.04c	1.33±0.02c	346.5±12.94c	336.0±12.36bA	373.8±7.68c	329.0±8.14b	10.0±0.61c	11.5±0.09b	10.5±0.54b	11.3±0.36b
	RT	1.42±0.03b	1.37±0.04b	1.46±0.03b	1.42±0.01bA	404.7±10.77b	342.9±15.06b	390.1±6.05b	339.2±20.67b	10.9±0.48b	12.4±1.17a	10.7±0.67b	12.1±0.32b
	NT	1.53±0.05a	1.50±0.05aA	1.53±0.04a	1.50±0.02aA	495.7±1.93a	436.7±17.17aA	484.2±22.56a	440.0±12.37a	11.2±0.63a	12.2±1.11a	11.2±0.81a	13.1±0.98a
20-30	PT	1.45±0.04b	1.30±0.04c	1.44±0.05b	1.32±0.01b	369.5±9.91c	314.45±9.80c	412.6±19.36c	313.5±14.34c	10.0±1.05b	11.1±0.12bA	12.1±0.82b	13.2±0.77bA
	RT	1.52±0.04a	1.48±0.02bA	1.54±0.06a	1.50±0.06aA	442.3±6.46b	439.0±15.86bA	511.0±0.56b	468.0±24.83b	10.4±0.57a	12.4±0.03a	12.4±0.43a	13.3±0.45bA
	NT	1.54±0.05a	1.53±0.01aA	1.55±0.03a	1.54±0.02aA	466.1±11.21a	471.0±18.89aA	541.0±2.37a	532.0±7.66aA	10.6±0.11a	12.9±0.86a	13.0±0.44a	14.4±0.55a
30-40	PT	1.53±0.03a	1.51±0.02aA	1.53±0.04a	1.48±0.06b	816.4±11.78a	772.8±9.95aA	854.0±14.2a	833.3±7.07aA	14.3±0.85b	15.0±0.28bA	13.3±1.09b	14.0±0.78bA
	RT	1.53±0.05a	1.52±0.04aA	1.55±0.01a	1.53±0.04aA	662.4±5.38b	657.4±22.10bA	718.9±24.63b	674.5±3.38bA	14.6±0.38b	15.1±0.77bA	13.3±0.29b	14.1±0.17bA
	NT	1.56±0.02a	1.55±0.05aA	1.57±0.03a	1.56±0.01aA	550.0±11.82c	520.2±17.37cA	590.0±6.12c	560.0±21.81c	15.2±0.22a	16.6±1.16a	15.0±0.17a	15.7±1.17aA
Analysis of variance													
T		***		***		ns		ns		*		**	
R		*		*		ns		ns		*		*	
T*R		ns		ns		ns		ns		ns		ns	

PT, RT and NT indicate plow-till, rotary-till and no-till, respectively. RR and RI indicate residue removal and residue incorporation, respectively. T and R indicate tillage and residue treatments, respectively. Values are expressed as the mean ± standard error. Different lowercase letters on mean values indicate significant differences at $P < 0.05$. Differences are significant at $P < 0.05$ between residue removal and residue incorporation under different tillage treatments except for figures marked A. *$P < 0.05$; **$P < 0.01$; ***$P < 0.001$; ns, not significant.

Supplementary Table S3. Soil organic carbon, total nitrogen and C:N ratio influenced by tillage and residue treatments at the silking stage of spring maize.

Depth (cm)	Treatment	Soil organic carbon (g kg^{-1})				Total nitrogen (g kg^{-1})				C:N ratio			
		2014		2015		2014		2015		2014		2015	
		RR	RI	RR	RI	RR	RI	RR	RI	RR	RI	RR	RI
0-10	PT	13.0±0.01c	13.2±0.07cA	13.1±0.07c	13.3±0.02cA	0.97±0.02b	0.99±0.01bA	0.99±0.03b	1.00±0.01bA	13.31±0.04c	13.33±0.05cA	13.29±0.05c	13.32±0.06c
	RT	13.7±0.03b	14.1±0.01b	13.8±0.04b	14.3±0.03b	1.02±0.03a	1.05±0.01a	1.03±0.03a	1.06±0.04a	13.42±0.01b	13.48±0.02b	13.43±0.04b	13.49±0.01b
	NT	14.0±0.06a	14.8±0.03a	14.4±0.03a	15.0±0.04a	1.03±0.01a	1.09±0.02a	1.06±0.04a	1.10±0.02a	13.51±0.01a	13.59±0.04a	13.52±0.01a	13.62±0.05a
10-20	PT	13.8±0.07b	13.8±0.08bA	14.0±0.03b	14.4±0.06b	1.02±0.01a	1.03±0.01aA	1.04±0.01a	1.07±0.01a	13.43±0.03a	13.45±0.01bA	13.44±0.04a	13.47±0.01b
	RT	13.9±0.01a	14.5±0.07a	14.1±0.02a	14.8±0.11a	1.04±0.02a	1.07±0.02ab	1.05±0.01a	1.09±0.01a	13.44±0.01a	13.50±0.02a	13.45±0.01a	13.52±0.04a
	NT	13.2±0.06c	13.4±0.05cA	13.2±0.10c	13.6±0.02c	0.98±0.01b	1.00±0.01bA	0.98±0.03b	1.01±0.02b	13.41±0.01b	13.44±0.05b	13.44±0.04a	13.50±0.04a
20-30	PT	14.1±0.01a	14.9±0.08a	14.3±0.06a	15.2±0.04a	1.05±0.01a	1.10±0.02a	1.06±0.02a	1.12±0.03a	13.50±0.03a	13.53±0.04a	13.51±0.01a	13.56±0.05a
	RT	13.3±0.07b	13.6±0.01bA	13.4±0.01b	13.6±0.05bA	0.99±0.03b	1.01±0.03bA	0.99±0.01b	1.01±0.04bA	13.44±0.04b	13.50±0.03b	13.50±0.02a	13.54±0.01a
	NT	12.7±0.02c	12.8±0.06cA	12.8±0.07c	13.0±0.07cA	0.94±0.01c	0.95±0.02cA	0.95±0.04c	0.96±0.04cA	13.46±0.02b	13.48±0.04bA	13.47±0.01b	13.51±0.02b
Analysis of variance													
T		ns		ns		ns		ns		ns		**	
R		*		*		*		*		*		*	
T*R		ns		ns		ns		ns		ns		ns	

PT, RT and NT indicate plow-till, rotary-till and no-till, respectively. RR and RI indicate residue removal and residue incorporation, respectively. T and R indicate tillage and residue treatments, respectively. Values are expressed as the mean ± standard error. Different lowercase letters on mean values indicate significant differences at $P < 0.05$. Differences are significant at $P < 0.05$ between residue removal and residue incorporation under different tillage treatments except for figures marked A. *$P < 0.05$; **$P < 0.01$; ***$P < 0.001$; ns, not significant.

Supplementary Table S4. Soil organic carbon, total nitrogen and C:N ratio influenced by tillage and residue treatments at the grain-filling stage of spring maize.

Depth (cm)	Treatment	Soil organic carbon (g kg^{-1})				Total nitrogen (g kg^{-1})				C:N ratio			
		2014		2015		2014		2015		2014		2015	
		RR	RI	RR	RI	RR	RI	RR	RI	RR	RI	RR	RI
0-10	PT	12.9±0.01c	13.6±0.04c	13.4±0.04c	13.7±0.07c	0.97±0.02b	1.02±0.03b	1.01±0.03b	1.02±0.03bA	13.36±0.04c	13.39±0.02cA	13.36±0.01c	13.42±0.01c
	RT	13.8±0.06b	14.2±0.02b	14.0±0.02b	14.5±0.02b	1.02±0.01a	1.05±0.01b	1.03±0.04b	1.07±0.01a	13.47±0.05b	13.49±0.01bA	13.49±0.05b	13.52±0.02b
	NT	14.2±0.04a	14.9±0.05a	14.6±0.07a	15.2±0.06a	1.04±0.03a	1.10±0.01a	1.08±0.02a	1.11±0.03a	13.57±0.02a	13.60±0.02a	13.54±0.03a	13.64±0.03a
10-20	PT	13.8±0.05b	13.9±0.04bA	14.1±0.08b	14.6±0.01b	1.02±0.01a	1.03±0.02bA	1.05±0.02aA	1.08±0.04a	13.47±0.01b	13.50±0.02a	13.48±0.05b	13.50±0.04bA
	RT	14.1±0.04a	14.6±0.07a	14.3±0.06a	15.0±0.01	1.04±0.01a	1.08±0.04a	1.06±0.04a	1.10±0.03a	13.51±0.02a	13.52±0.01aA	13.56±0.06a	13.59±0.05aA
	NT	13.2±0.07c	13.6±0.05cA	13.4±0.02c	13.8±0.01c	0.99±0.03b	1.01±0.04bA	0.99±0.04b	1.02±0.02b	13.41±0.04c	13.46±0.04b	13.44±0.03c	13.47±0.05c
20-30	PT	14.3±0.05a	15.0±0.03a	14.3±0.01a	15.3±0.02a	1.06±0.03a	1.11±0.03a	1.06±0.02a	1.12±0.01a	13.53±0.06a	13.55±0.01a	13.58±0.04a	13.61±0.05a
	RT	13.4±0.07b	13.8±0.03b	13.5±0.06b	13.8±0.05bA	1.00±0.02b	1.02±0.01bA	1.00±0.03b	1.02±0.03bA	13.44±0.05b	13.46±0.02bA	13.56±0.04a	13.57±0.03bA
	NT	12.7±0.02c	13.0±0.02cA	12.9±0.04c	13.1±0.02cA	0.94±0.01c	0.96±0.01cA	0.95±0.01c	0.96±0.03cA	13.51±0.03a	13.53±0.01aA	13.53±0.06b	13.56±0.05b
Analysis of variance													
T		ns		ns		ns		ns		ns		**	
R		**		**		*		*		*		*	
T*R		ns		ns		ns		ns		ns		ns	

PT, RT and NT indicate plow-till, rotary-till and no-till, respectively. RR and RI indicate residue removal and residue incorporation, respectively. T and R indicate tillage and residue treatments, respectively. Values are expressed as the mean ± standard error. Different lowercase letters on mean values indicate significant differences at $P < 0.05$. Differences are significant at $P < 0.05$ between residue removal and residue incorporation under different tillage treatments except for figures marked A. *$P < 0.05$; **$P < 0.01$; ***$P < 0.001$; ns, not significant.

Supplementary Table S5. Root diameter, root-length density and root surface area density influenced by tillage and residue treatments at the silking stage of spring maize.

Treatment	Root diameter (mm)				Root-length density (cm cm^{-3})				Root surface area density (cm^{2} cm^{-3})			
	2014		2015		2014		2015		2014		2015	
	RR	RI	RR	RI	RR	RI	RR	RI	RR	RI	RR	RI
PT	6.25±0.43b	6.73±0.54b	7.14±0.37b	7.65±0.42b	5.88±0.48b	6.04±0.53b	6.78±0.59b	7.17±0.54b	1.32±0.40b	1.51±0.46b	1.30±0.42b	1.45±0.46b
RT	6.35±0.52b	7.10±0.60b	7.51±0.40b	7.86±0.69b	5.64±0.49b	5.97±0.52b	6.67±0.58b	7.04±0.61b	1.43±0.42a	1.53±0.44a	1.43±0.44a	1.56±0.46a
NT	7.03±0.69a	7.57±0.78a	7.98±0.51a	8.23±0.90a	5.47±0.50a	5.82±0.53a	6.54±0.58a	6.85±0.55a	1.46±0.44a	1.56±0.47a	1.46±0.42a	1.60±0.47a
Analysis of variance												
T	**		*		ns		ns		ns		***	
R	***		*		*		*		*		***	
T*R	ns		ns		ns		ns		ns		ns	

PT, RT and NT indicate plow-till, rotary-till and no-till, respectively. RR and RI indicate residue removal and residue incorporation, respectively. T and R indicate tillage and residue treatments, respectively. Values are expressed as the mean ± standard error. Different lowercase letters on mean values indicate significant differences at $P < 0.05$. Differences are significant at $P < 0.05$ between residue removal and residue incorporation under different tillage treatments except for figures marked A. *$P < 0.05$; **$P < 0.01$; ***$P < 0.001$; ns, not significant.

Supplementary Table S6. Root diameter, root-length density and root surface area density influenced by tillage and residue treatments at the grain-filling stage of spring maize.

Treatment	Root diameter (mm)				Root-length density (cm cm^{-3})				Root surface area density (cm^{2} cm^{-3})			
	2014		2015		2014		2015		2014		2015	
	RR	RI	RR	RI	RR	RI	RR	RI	RR	RI	RR	RI
PT	4.78±0.20b	5.13±0.22b	5.16±0.27b	5.64±0.29b	4.13±0.36b	4.97±0.43b	5.57±0.48a	6.43±0.56a	1.06±0.29b	1.21±0.33b	1.20±0.38b	1.30±0.42c
RT	4.84±0.23b	5.18±0.25b	5.21±0.29b	5.73±0.31b	4.07±0.36b	4.89±0.43b	5.36±0.46a	6.24±0.53b	1.12±0.28a	1.23±0.33a	1.24±0.35a	1.36±0.39b
NT	5.55±0.33a	5.75±0.33a	5.59±0.39a	6.04±0.43a	3.89±0.34a	4.86±0.42a	4.86±0.43c	5.81±0.52b	1.17±0.31a	1.25±0.33a	1.34±0.37a	1.39±0.42a
Analysis of variance												
T	***		*		ns		**		ns		***	
R	*		***		***		***		***		***	
T*R	ns		ns		ns		ns		ns		***	

PT, RT and NT indicate plow-till, rotary-till and no-till, respectively. RR and RI indicate residue removal and residue incorporation, respectively. T and R indicate tillage and residue treatments, respectively. Values are expressed as the mean ± standard error. Different lowercase letters on mean values indicate significant differences at $P < 0.05$. Differences are significant at $P < 0.05$ between residue removal and residue incorporation under different tillage treatments except for figures marked A. *$P < 0.05$; **$P < 0.01$; ***$P < 0.001$; ns, not significant.

Supplementary Table S7. Root biomass, shoot biomass and root-shoot ratio influenced by tillage and residue treatments at the silking stage of spring maize.

Treatment	Root biomass (g plant^{-1})				Shoot biomass (g plant^{-1})				Root-shoot ratio			
	2014		2015		2014		2015		2014		2015	
	RR	RI	RR	RI	RR	RI	RR	RI	RR	RI	RR	RI
PT	16.8±1.85b	21.2±2.34b	16.7±0.19b	20.3±0.06b	132.7±14.60a	133.0±4.91aA	124.7±7.06b	132.0±4.98b	0.126±0.001b	0.160±0.021b	0.134±0.003b	0.154±0.004b
RT	18.5±2.03b	23.2±2.55b	19.7±0.32b	24.7±0.06b	126.0±7.51a	127.9±5.53aA	124.8±6.85b	139.2±2.16b	0.146±0.010a	0.181±0.013a	0.158±0.006a	0.177±0.002a
NT	20.2±2.22a	23.8±2.62a	20.8±0.16a	26.5±1.30a	122.0±13.42b	125.3±13.78bA	125.7±0.15a	143.8±1.25a	0.165±0.001a	0.190±0.001a	0.165±0.001a	0.184±0.011a
Analysis of variance												
T	*		***		ns		ns		***		***	
R	**		***		ns		***		***		***	
T*R	ns		*		ns		ns		ns		ns	

PT, RT and NT indicate plow-till, rotary-till and no-till, respectively. RR and RI indicate residue removal and residue incorporation, respectively. T and R indicate tillage and residue treatments, respectively. Values are expressed as the mean ± standard error. Different lowercase letters on mean values indicate significant differences at $P < 0.05$. Differences are significant at $P < 0.05$ between residue removal and residue incorporation under different tillage treatments except for figures marked A. *$P < 0.05$; **$P < 0.01$; ***$P < 0.001$; ns, not significant.

Supplementary Table S8. Root biomass, shoot biomass and root-shoot ratio influenced by tillage and residue treatments at the grain-filling stage of spring maize.

Treatment	Root biomass (g plant^{-1})				Shoot biomass (g plant^{-1})				Root-shoot ratio			
	2014		2015		2014		2015		2014		2015	
	RR	RI	RR	RI	RR	RI	RR	RI	RR	RI	RR	RI
PT	15.0±1.65b	19.4±0.13b	16.0±0.14b	18.6±0.28b	344.3±37.87a	350.5±18.59bA	330.6±20.09b	350.3±4.85b	0.044±0.001b	0.055±0.003b	0.048±0.004b	0.053±0.009b
RT	16.6±0.96b	20.1±0.21b	17.5±0.95b	22.2±1.44b	333.4±36.67b	360.4±22.00b	340.6±10.46b	374.6±14.91b	0.050±0.005a	0.056±0.003a	0.051±0.003a	0.059±0.007a
NT	18.8±2.07a	21.6±0.38a	20.5±1.06a	24.1±0.18a	336.5±37.02b	373.7±25.35a	368.4±61.29a	393.1±17.87a	0.056±0.001a	0.058±0.003a	0.056±0.008a	0.061±0.010a
Analysis of variance												
T	**		***		ns		ns		***		***	
R	***		***		ns		***		***		**	
T*R	ns		ns		ns		ns		*		**	

PT, RT and NT indicate plow-till, rotary-till and no-till, respectively. RR and RI indicate residue removal and residue incorporation, respectively. T and R indicate tillage and residue treatments, respectively. Values are expressed as the mean ± standard error. Different lowercase letters on mean values indicate significant differences at $P < 0.05$. Differences are significant at $P < 0.05$ between residue removal and residue incorporation under different tillage treatments except for figures marked A. *$P < 0.05$; **$P < 0.01$; ***$P < 0.001$; ns, not significant.
